# Social Support and Cognition: A Systematic Review

**DOI:** 10.3389/fpsyg.2021.637060

**Published:** 2021-02-23

**Authors:** Stefanella Costa-Cordella, Camilo Arevalo-Romero, Francisco J. Parada, Alejandra Rossi

**Affiliations:** ^1^Centro de Estudios en Neurociencia Humana y Neuropsicología, Facultad de Psicología, Universidad Diego Portales, Santiago, Chile; ^2^Centro de Estudios en Psicología Clínica y Psicoterapia, Facultad de Psicología, Universidad Diego Portales, Santiago, Chile; ^3^Programa de Magíster en Neurociencia Social, Facultad de Psicología, Universidad Diego Portales, Santiago, Chile

**Keywords:** social support, cognition, social interaction, cognitive performance, cognitive functioning

## Abstract

Although the influence of social support in health is a widely acknowledged factor, there is a significant gap in the understanding of its role on cognition. The purpose of this systematic review was, therefore, to determine the state-of-the-art on the literature testing the association between social support and cognition. Using six databases (WoS, PubMed, ProQuest, PsycINFO, Scopus and EBSCOhost), we identified 22 articles published between 1999 and 2019 involving an empirical quantitative focus which meet the inclusion criteria. Data extraction was performed following PRISMA recommendations. To summarize the extracted data, we used a narrative synthesis approach. Despite limitations, there is overall preliminary evidence of a relevant positive association between social support and cognition. Our results demonstrate there is enough information for an outbreak of experimental research in the area and an expansion of this body of knowledge. We argue that the present evidence lays the foundations for a more comprehensive theoretical model, one that corresponds with the complexity of the topic and possibly considers models derived from social interaction and active inference theories.

## Introduction

Human beings are defined by and within their social environment. Meaningful social interaction -and cultural learning emerging within those interactions- play a key role in the development and enactment of cognitive acts (Di Paolo and De Jaegher, 2012; Heyes, 2020). Early in life, our relationships have the power to shape us and our surroundings (Keverne and Curley, 2008; Roth and David Sweatt, 2011). Infant temperament potentially shows this dynamicity as it shapes the family environment (Caspi and Shiner, 2008; Kiff et al., 2011), which in turn, modulates the kind of interactions the baby is exposed to (Brackbill et al., 1990; Saudino, 2005; Bates et al., 2019). Early social interactions have been shown to have an essential ontogenetic role (Papoušek and Papoušek, 1989; Bråten, 1998; Oster, 2004; Rojas-Líbano and Parada, 2019) to the extent that the quality of first meaningful interactions is strongly associated with overall health outcomes in life (Miller and Chen, 2010; Miller et al., 2011; Pietromonaco et al., 2013). Indeed, social relationships are powerful enough that their perceived quality in a person's life is comparable with standard risk factors such as smoking, blood pressure, and physical activity (Uchino et al., 1996, 2018).

Social support can be understood as any resource that flows through and from social relationships (Waite, 2018). These relationships are based on social interactions and could be virtual, implied, imagined, real, momentary and/or ongoing. From a health science perspective, social support is conceived as the available support for an individual through social ties with other people, groups, or the community in general (Ozbay et al., 2008). Again, the available support could be virtual, implied, imagined, real, transitory and/or continuing. Social support is often studied in terms of instrumental support, emotional support, advice or information, financial support, provision of care, moral support, and social connections to others (Waite, 2018). Social support has been extensively studied in the last decades (Uchino, 2004; Gottlieb and Bergen, 2010). Longitudinal studies on the effect of perceived social support on health outcomes show that having significant companionship reduces the risk of heart disease and cardiovascular incidents (Anthony and O'Brien, 1999; Havranek et al., 2015; Ginting et al., 2016), respiratory diseases (Cohen et al., 2015; Janicki Deverts et al., 2017) and strokes (Valtorta et al., 2016) among other pathologies (e.g., treatment outcomes in breast cancer; Hinzey et al., 2016). Social support even seems to impact mortality rates. A recent meta-analysis of 148 independent studies indicates that social relationships are significant predictors of mortality, revealing a robust effect of social support on longevity and overall satisfaction with life (Holt-Lunstad et al., 2010, 2015).

Evidence from experimental approaches shows that the presence of a supportive figure (i.e., romantic partner or family member) has analgesic effects (Goldstein et al., 2018), stimulates facial expressivity (Frith, 2009; Vervoort et al., 2011; Karmann et al., 2014; Gallant and Hadjistavropoulos, 2017), modulates physiological responses (Uchino et al., 2012; Bowen et al., 2014) and protects participants against the deleterious effects of stress (Heinrichs et al., 2003; Ditzen et al., 2006; Cosley et al., 2010; Meuwly et al., 2012; McQuaid et al., 2016; Janicki Deverts et al., 2017). In the long term, social support has been shown to influence cardiovascular reactivity (Fontana et al., 1999; Uno et al., 2002; Lett et al., 2005) and the activity of the hypothalamic-pituitary-adrenocortical (HPA) axis (Hostinar et al., 2014; Kirsch and Lehman, 2015). The latter is significant in that it identifies a social-interactional component as a damping factor of the HPA axis response to stressors. This effect might be linked in a mechanistic sense, to the oxytocinergic systems and prefrontal neural networks. These systems may act as putative biological mediators based on the benefits of social support (Heinrichs et al., 2003; Ditzen et al., 2006; McQuaid et al., 2016). Taking into account the role of oxytocinergic systems and prefrontal networks in socio-cognitive processes, the effect of this social-interactional component has a particular biological relevance (Ross and Young, 2009; Guastella and MacLeod, 2012; Mitre et al., 2016).

Consistent with these assumptions, several studies note the relevance of social support on cognitive performance. Higher levels of social support have been frequently associated with better cognitive functioning and less cognitive decline (Seeman et al., 2001; Kelly et al., 2017), while social isolation shows the opposite pattern (Cacioppo and Hawkley, 2009; Yin et al., 2019). Importantly, the neural dynamics involved in both social behavior and cognition are seen in social interactions (Di Paolo and De Jaegher, 2012, 2015; Mende-Siedlecki et al., 2013; De Jaegher et al., 2016; Redcay and Schilbach, 2019). For example the mentalizing and the mirror neural systems have been described extensively in the literature (Dunbar, 2014; Bzdok and Dunbar, 2020). These -among other neural networks- perform key roles in producing adaptive social behavior.

From contemporary perspectives on cognition (Gallagher, 2001; Robbins and Aydede, 2001; Wilson and Clark, 2001; Newen et al., 2018; Parada and Rossi, 2018), the implied, imagined, and/or actual social interaction should have a central role in the development and operation of cognitive processes, both being determined by and determining the dynamic agent/environment relationship. As an example, the fairly recent *interactional brain hypothesis* (Di Paolo and De Jaegher, 2012) considers interaction with others (may it be virtual, implied, imagined, real, momentary and/or continuing) to be a constitutive variable of brain development and cognitive processes. Therefore, interaction as a dimension of social support, may play a crucial role in the emergence of cognition (Heyes, 2020). However, what is known about the relationship between social support and cognition comes from observational studies using self-report measures. In turn, cognition has been mostly tested through psychometric tests with little or no consideration of the context in which cognition emerges. Thus, the dominant perspective of cognition across these studies could be interpreted as internalist (i.e., mental abilities are constituted by the intrinsic operational properties of spatially-localizable neurocognitive structures). This is evident in the theoretical framework and result interpretation of the studies, which rely on computational/representationalist conceptions of mind (i.e., using concepts like memory or attention, which are understood as abstract processes occurring solely inside an agent's mind).

As noted, the role of social support on cognition has rarely been studied from an experimental or longitudinal design. This has hindered the extraction of causal inferences about this relationship. One of the reasons for such paucity of research may be the lack of a unifying model for understanding the role of social support on cognition. Therefore, the first step toward filling this gap entails determining the current state of literature on this topic (i.e., last decades). Accordingly, the present work strove to achieve this by systematically reviewing the scientific literature on the relationship between social support and cognition through three specific aims:

- To evaluate how social support has been measured in the literature between 1999 and 2019
- To assess how cognition has been measured in the literature between 1999 and 2019
- To describe the reported relationships between social support and cognition in the reviewed literature.

## Methods

The present systematic review was conducted according to the Preferred Reporting Items for Systematic Reviews and Meta-Analyses (PRISMA, Moher et al., 2009). The methods were outlined in an initial protocol which specified the review's interests, objectives, and methods, currently available in the Open Science Framework (OSF, https://osf.io/hn7mc/).

### Eligibility Criteria

The main focus of this review was to identify empirical quantitative studies that explored the association between social support and cognition.

The inclusion criteria were

- Articles written in English
- Published in peer-reviewed journals between 1999 and 2019
- Observational or experimental studies that analyzed the relationship between social support and cognitive performance
- Human participants only.

Studies were excluded if

- They were mainly a review, a qualitative study, an intervention, or a psychometric study
- They were considered to be “gray literature” (conference abstracts and proceedings, unpublished data, preprints, government publications and reports, dissertations and theses, among others).

### Information Sources

For the advanced search, six electronic databases were used: WoS, PubMed, ProQuest, PsycINFO, Scopus and EBSCOhost. After the full-text review, 22 articles met the inclusion criteria. The search was run from January 1999 to July 2019.

### Search Strategy

The search terms were: “social support” AND cognitive OR cognition, and the search fields were title and summary.

### Study Selection

**Figure 1:**
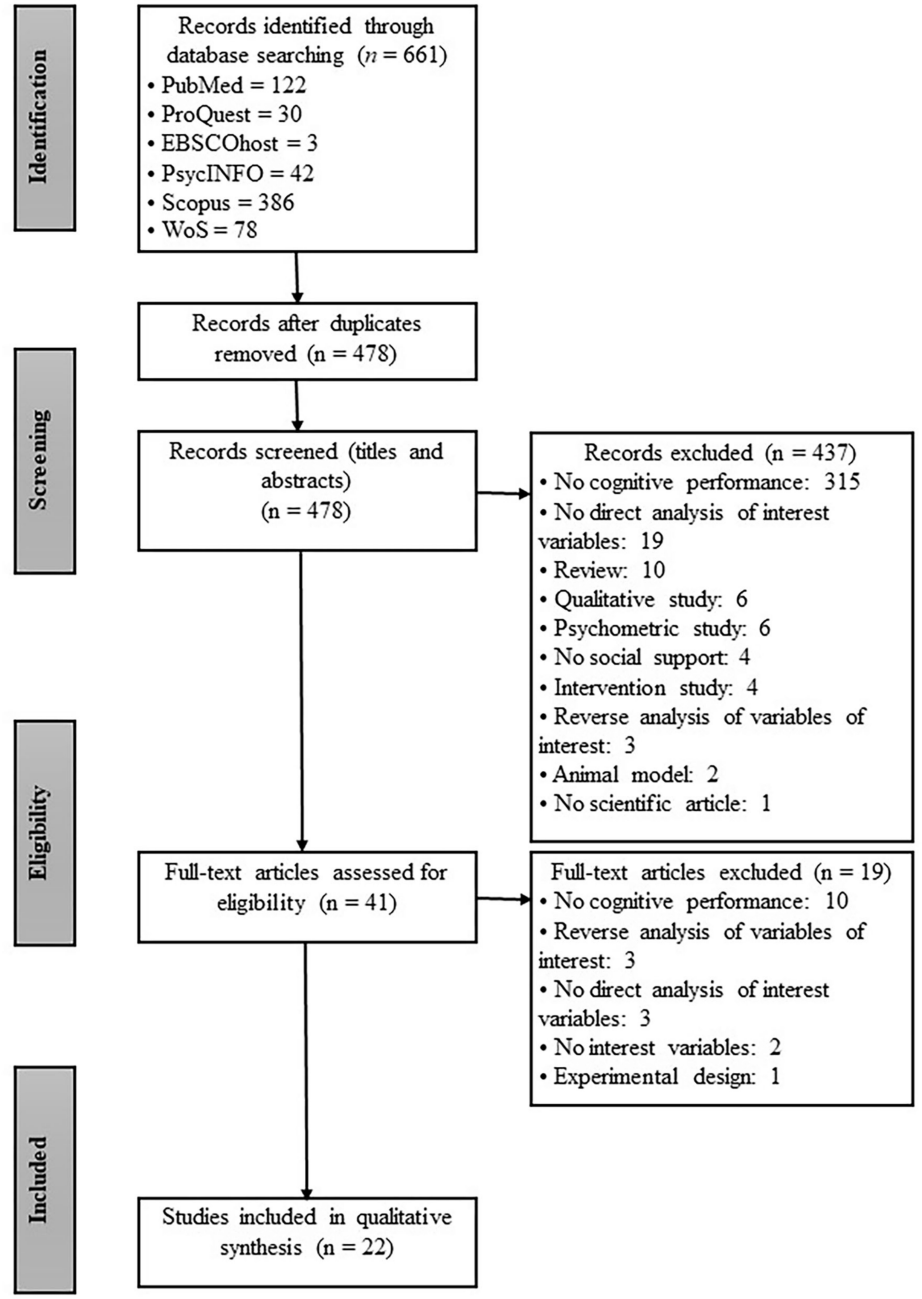
Flow diagram based on PRISMA guidelines.

The selection process is represented schematically in Figure 1, which details the total number of articles found, the number of articles after removing duplicates and those that did not meet the inclusion criteria, and the total number of articles selected for further analysis. The initial search identified 661 articles. Author SCC conducted the advanced search in the databases. Once all outputs were compiled from the databases in Microsoft Office Excel, author CAR eliminated duplicates, leaving 478 articles. These 478 studies were divided amongst authors SCC and CAR to independently screen titles and abstracts against the inclusion criteria. There were 38 discrepancies resolved through author AR mediation. At this stage, 41 articles were selected for full-text review between SCC (34%), CAR (34%) and AR (32%). After the full-text review, 22 articles met the inclusion criteria and were included in the review. For more details on the whole process and access to the list of excluded studies, you can also visit the database of this process in OSF (https://osf.io/wbzfk/).

### Data Extraction and Analysis

Data extraction was first performed independently by authors SCC, CAR, and AR on seven articles to cross-check and polish the process. These articles were the same for each author. Next, each author extracted data from the remaining articles separately [SCC (six), CAR (six) and AR (three)].The full text of the articles was read, exploring their methodological characteristics and results. Information on the study's design, sample size, measures used, was recorded (see **Table 3**). Additionally, results on the relationship between social support and cognition were noted as presented in **Tables 4**, **6**. A risk of bias analysis was carried out for each individual study as well and summary of risk of bias analysis using the Quality Assessment Tool for Observational Cohort and Cross-Sectional Studies (National Heart, Lung, and Blood Institute, 2019). The summary of the PRISMA checklist for the present systematic review is presented in Table 1.

**Table 1:** PRISMA checklist.

**Section/topic**	**N^**°**^**	**Checklist item**	**Reported on section**
**Title**			
Title	1	Identify the report as a systematic review, meta-analysis, or both.	Title
**Abstract**			
Structured summary	2	Provide a structured summary including, as applicable: background; objectives; data sources; study eligibility criteria, participants, and interventions; study appraisal and synthesis methods; results; limitations; conclusions and implications of key findings; systematic review registration number.	Abstract
**Introduction**			
Rationale	3	Describe the rationale for the review in the context of what is already known.	Introduction
Objectives	4	Provide an explicit statement of questions being addressed with reference to participants, interventions, comparisons, outcomes, and study design (PICOS).	Introduction
**Methods**			
Protocol and registration	5	Indicate if a review protocol exists, if and where it can be accessed (e.g., Web address), and, if available, provide registration information including registration number.	Methods
Eligibility criteria	6	Specify study characteristics (e.g., PICOS, length of follow-up) and report characteristics (e.g., years considered, language, publication status) used as criteria for eligibility, giving rationale.	Methods
Information sources	7	Describe all information sources (e.g., databases with dates of coverage, contact with study authors to identify additional studies) in the search and date last searched.	Methods
Search	8	Present full electronic search strategy for at least one database, including any limits used, such that it could be repeated.	Methods
Study selection	9	State the process for selecting studies (i.e., screening, eligibility, included in systematic review, and, if applicable, included in the meta-analysis).	Methods
Data collection process	10	Describe method of data extraction from reports (e.g., piloted forms, independently, in duplicate) and any processes for obtaining and confirming data from investigators.	Methods
Data items	11	List and define all variables for which data were sought (e.g., PICOS, funding sources) and any assumptions and simplifications made.	Methods
Risk of bias in individual studies	12	Describe methods used for assessing risk of bias of individual studies (including specification of whether this was done at the study or outcome level), and how this information is to be used in any data synthesis.	-
Summary measures	13	State the principal summary measures (e.g., risk ratio, difference in means).	-
Synthesis of results	14	Describe the methods of handling data and combining results of studies, if done, including measures of consistency (e.g., I^2^) for each meta-analysis.	Methods
Risk of bias across studies	15	Specify any assessment of risk of bias that may affect the cumulative evidence (e.g., publication bias, selective reporting within studies).	-
Additional analyses	16	Describe methods of additional analyses (e.g., sensitivity or subgroup analyses, meta-regression), if done, indicating which were pre-specified.	-
**Results**			
Study selection	17	Give numbers of studies screened, assessed for eligibility, and included in the review, with reasons for exclusions at each stage, ideally with a flow diagram.	Results
Study characteristics	18	For each study, present characteristics for which data were extracted (e.g., study size, PICOS, follow-up period) and provide the citations.	Results
Risk of bias within studies	19	Present data on risk of bias of each study and, if available, any outcome level assessment (see item 12).	-
Results of individual studies	20	For all outcomes considered (benefits or harms), present, for each study: (a) simple summary data for each intervention group (b) effect estimates and confidence intervals, ideally with a forest plot.	Results
Synthesis of results	21	Present results of each meta-analysis done, including confidence intervals and measures of consistency.	-
Risk of bias across studies	22	Present results of any assessment of risk of bias across studies (see Item 15).	-
Additional analysis	23	Give results of additional analyses, if done (e.g., sensitivity or subgroup analyses, meta-regression [see Item 16]).	-
**Discussion**			
Summary of evidence	24	Summarize the main findings including the strength of evidence for each main outcome; consider their relevance to key groups (e.g., healthcare providers, users, and policy makers).	Discussion
Limitations	25	Discuss limitations at study and outcome level (e.g., risk of bias), and at review-level (e.g., incomplete retrieval of identified research, reporting bias).	Discussion
Conclusions	26	Provide a general interpretation of the results in the context of other evidence, and implications for future research.	Discussion
**Funding**			
Funding	27	Describe sources of funding for the systematic review and other support (e.g., supply of data); role of funders for the systematic review.	Funding

### Analytic Approach Concerning the Synthesis of Findings

A narrative synthesis approach (Popay et al., 2006; Ryan, 2013) was used to summarize the extracted data. This method provides a framework to analyze possible data associations. We used our specific aims as the main summary step (i.e., 1-To evaluate measures of social support; 2- To assess measures of cognition; 3-To describe the reported relationships between social support and cognition across the studies). Next, studies were grouped according to the types of instruments used (for aims one and two) and by participant ages for aim three.

## Results

Data from 22 selected articles were divided into two tables (**Tables 3**, **4**). **Table 3** contains the name of the main author and publication year, sample number, percentage of women and men in the sample, population, location, ethnic group, age range, age group, social support measures, and cognition measures. **Table 4** contains the following categories: author and study design; analysis method; main results.

### Risk of Bias

Studies were assessed for risk of bias using the Quality Assessment Tool for Observational Cohort and Cross-Sectional Studies (National Heart, Lung, and Blood Institute, 2019) as presented in Table 2. This tool has been used in previous reviews (Harris et al., 2016; Koppen et al., 2016; Connolly et al., 2017; Carbia et al., 2018; Amit et al., 2020). Using this instrument the quality of the research manuscripts included in the present review was evaluated. From the 22 articles, a trend is seen in the reporting of sample size justification (criteria 5). Only 20% of the selected studies reported how the sample size was calculated. This challenges representativity of the samples as only 25% of the studies reported the participation rate of eligible participants. The 72.7% use a cross-sectional design which also results in an increase in bias due to the nature of the design itself, where causation cannot be established. A strength of the selected articles is that 95.45% used valid and reliable instruments for measuring cognition and a 90.9% used valid and reliable measures of social support. However, this may not reflect how properly social support and cognition are measured in these studies. Only a small number of articles define social support and/or cognition clearly and they generally use self report measures that may not accurately reflect the experience of social support or the cognitive phenomena. These issues are examined in the Discussion section.

**Table 2:** Summary of risk of bias.

	**Criteria**														
**References**	**1**	**2**	**3**	**4**	**5**	**6**	**7**	**8**	**9**	**10**	**11**	**12**	**13**	**14**	**Quality Rating**
Seeman et al. (2001)	Yes	Yes	Yes	Yes	Yes	NA	Yes	NA	Yes	Yes	Yes	NA	Yes	Yes	Good
Yeh and Liu (2003)	Yes	Yes	No	Yes	Yes	NA	No	NA	Yes	No	Yes	NA	NA	Yes	Good
Whitfield and Wiggins (2003)	Yes	Yes	NR	Yes	Yes	NA	No	NA	Yes	No	Yes	NA	NA	Yes	Fair
Slykerman et al. (2005)	Yes	Yes	NR	Yes	No	NA	Yes	NA	Yes	Yes	Yes	NA	NA	Yes	Good
Dickinson et al. (2011)	Yes	Yes	NR	Yes	No	NA	Yes	NA	Yes	Yes	Yes	NA	NR	Yes	Good
Sims et al. (2011)	Yes	Yes	NR	Yes	No	NA	No	NA	Yes	No	Yes	NA	NA	Yes	Good
Zhu et al. (2012)	Yes	Yes	NR	Yes	No	NA	NA	NA	Yes	NA	Yes	NA	NA	Yes	Fair
Zuelsdorff et al. (2013)	Yes	Yes	NR	Yes	NR	NA	No	NA	Yes	Yes	Yes	NA	NA	Yes	Good
Ellwardt et al. (2013)	Yes	Yes	Yes	Yes	Yes	NA	NA	NA	Yes	NA	Yes	NA	NA	Yes	Good
Tanzer et al. (2013)	Yes	Yes	NR	Yes	No	No	Yes	No	Yes	No	Yes	NA	NA	Yes	Good
Ayotte et al. (2013)	Yes	Yes	NR	Yes	No	NA	NA	NA	NA	NA	NA	NA	NA	Yes	Good
Sims et al. (2014)	Yes	Yes	NR	Yes	No	NA	NA	NA	Yes	NA	Yes	NA	NA	Yes	Good
Pillemer and Holtzer (2015)	Yes	Yes	NR	Yes	No	NA	No	NA	Yes	No	Yes	NA	NA	Yes	Good
Yilmaz et al. (2015)	Yes	Yes	NR	Yes	No	NA	NA	NA	Yes	NA	Yes	NA	NA	No	Fair
Kats et al. (2016)	Yes	Yes	Yes	Yes	No	NA	Yes	NA	Yes	Yes	Yes	NA	NR	Yes	Good
Liao et al. (2016)	Yes	Yes	Yes	Yes	No	NA	Yes	NA	Yes	Yes	Yes	NA	NR	Yes	Good
Frith and Loprinzi (2017)	Yes	Yes	NR	Yes	No	NA	No	NA	No	No	Yes	NA	NA	Yes	Fair
La Fleur and Salthouse (2017)	Yes	Yes	NR	Yes	No	NA	No	NA	Yes	No	Yes	NA	NA	Yes	Good
Zuelsdorff et al. (2017)	Yes	Yes	NR	Yes	No	NA	No	NA	Yes	Yes	Yes	NA	NA	Yes	Good
Ge et al. (2017)	Yes	Yes	Yes	Yes	No	NA	NA	NA	Yes	No	Yes	NA	NA	Yes	Good
Zamora-Macorra et al. (2017)	Yes	Yes	NR	Yes	No	NA	NA	NA	Yes	No	Yes	NA	NA	Yes	Good
Wang et al. (2017)	Yes	Yes	NR	Yes	No	NA	NA	NA	Yes	NA	Yes	NA	NA	Yes	Good

*NA, not applicable; NR, not reported; CD, cannot determine; NHLBI, National Heart, Lung, and Blood Institute; You can see the criteria in the following link https://www.nhlbi.nih.gov/health-topics/study-quality-assessment-tools*.

### Study Characteristics

#### General Characteristics of Selected Studies

**Figure 2:**
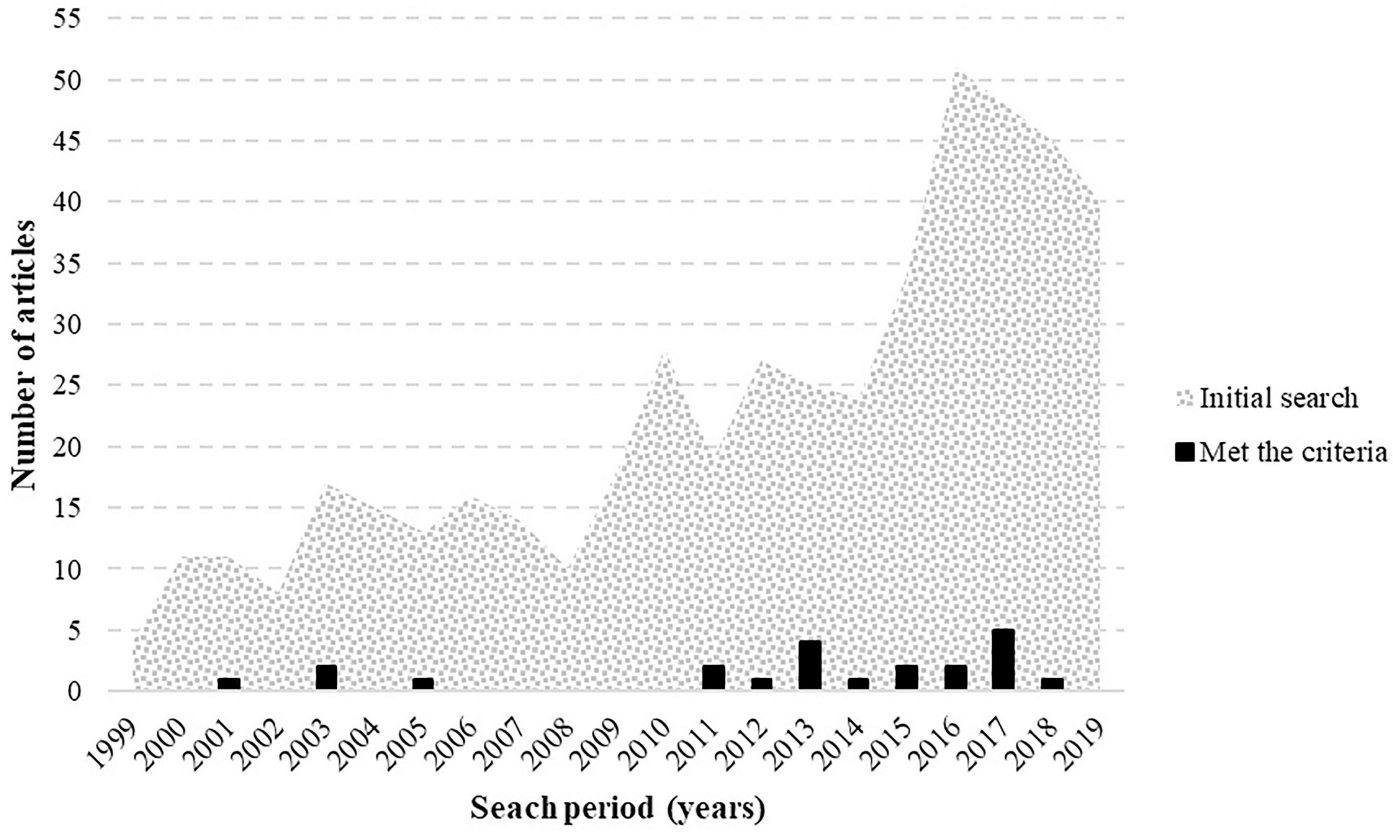
Histogram showing the frequency of articles on social support and cognition published during the period comprehended between 1999 and 2019. The textured area represents published articles on the topic while the black bars represents articles that met the inclusion/exclusion criteria and therefore were selected for the present review.

The search for scientific literature was carried out considering articles published between 1999 and 2019. The initial search (removing duplicate articles) found 137 studies (29%) between 1999 and 2009, and 359 (75%) between 2009 and 2019. The selected articles were published between 2001 and 2017. In this period, four articles (18%) were published between 2001 and 2009 and eighteen (82%) between 2009 and 2017 (Figure 2).

Of the 22 articles that met the criteria, 10 studies analyzed data collected in a larger sample (more than 1.000). Five studies assessed social support by asking questions in a non-standardized manner. All studies employed standardized tests to measure cognition. Detailed information is provided in Table 3.

**Table 3:** Overview of studies characteristics.

**References**	***n***	**Sex**	**Population**	**Location**	**Reported ethnic group**	**Age range**	**Age group**	**Social Support measures**	**Cognition measures**
Seeman et al. (2001)	1,145	♂: 45% ♀: 55%	No clinic	United States	African American: 20% White: 80%	70–79 M: 74 SD: 2.7	Older adults	MAB	18I-BNT; 4I-WAIS-R
Yeh and Liu (2003)	4,989	♂: 53% ♀: 47%	No clinic	Taiwan	NR	≥65 M: 73 SD: 5.49	Older adults	No, series of questions	SPMSQ
Whitfield and Wiggins (2003)	249	♂: 38% ♀: 62%	No clinic	United States	African American	47–91 M: 67.8 SD: 8.47	Older adults	NSBA	EPT
Slykerman et al. (2005)	550	NR	No clinic	New Zealand	NR	3 1/2	Children's	FSS	SBIS-4E
Dickinson et al. (2011)	213	♂ D: 44% ♀ D: 56% ♂ ND: 30% ♀ ND: 70%	Depressed (D) Non depressed (ND)	United States	Caucasian: 88.26% NR: 12%	≥60 D: M: 68.69 ND: M: 70.46	Older adults	DSSI	CERAD: MMSE, LT, CP, VLM; LM-WMS-R; WAIS-R: TMT-A, TMT-B, SDMT, DSF; DSB; ADS; DOT
Sims et al. (2011)	139	♂: 48% ♀: 52%	No clinic	United States	African American	M: 46 SD: 11.56	Middle-aged	ISEL	WCST; SC-WT
Zhu et al. (2012)	120	♂: 63% ♀: 37%	No clinic	China	NR	60 to >80 M: 71	Older adults	MSPSS	MMSE
Zuelsdorff et al. (2013)	623	♂: 29% ♀: 71%	No clinic	United States	NR	40–73 M: 56.7 SD: 6.5	Middle-aged Older adults	MOS	RAVLT; WAIS-III: DF, DB, LNS, TA, TB, SC-WT
Ellwardt et al. (2013)	2,255	♂: 46% ♀: 54%	No clinic	Netherlands	NR	55–85 M: 63.45 SD: 6.65	Older adults	No, series of questions	MMSE; RCPM
Tanzer et al. (2013)	142	♀: 100%	No clinic	Israel	NR	19–26 M: 23.22 SD: 1.31	Young adults	NRI	CT
Ayotte et al. (2013)	602	♂: 25% ♀: 75%	No clinic	NR	African American	48–90 M: 69 SD: 9.74	Older adults	Two scales based on the NSBA	HVLT; RAVLT; IRT; AST; BDST; OST; SILVMT; II-IV-ETS-VT; NCT; DST; IPT; SILSAT; LST*
Sims et al. (2014)	175	♂: 55% ♀: 45%	No clinic	United States	White: 87.7% African american: 9.9% Other: 2.4%	54–83 M: 66 SD: 6.92	Older adults	ISEL	SC-WT; JLO; WAIS-R: BD, DSF, DSB, VSF, VSB; WMS-R: VR-I, VR-II, LM-I, LM-II; TT
Pillemer and Holtzer (2015)	355	♂: 45% ♀: 55%	No clinic	United States	Caucasian: 87.3% NR: 13%	65–95 M: 77 SD: 6.94	Older adults	MOS-SSS	RBANS
Yilmaz et al. (2015)	121	♂: 43% ♀: 57%	Diabete mellitus	Turkey	NR	18–75 M: 57	Young adults Adults Older adults	MSPSS	SMMSE
Kats et al. (2016)	13,119	♂: 44% ♀: 56%	Atherosclerosis risk	United States	NR	45–64	Middle-aged Older adults	ISEL-SF; LSNS	DSST; DWRT; WFT
Liao et al. (2016)	6,863	♂: 71% ♀: 29%	NR	England	White: 92.3% NR: 8%	M: 55.8 SD: 6.03	Older adults	CPQ	AH4-I; PVF; SVF; 20-WAL
Frith and Loprinzi (2017)	1,874	♂: 41% ♀: 59%	No clinic	United States	Hispanic white: 83.4% NR: 17%	60–85 M: 70	Older adults	No, series of questions	DSST
La Fleur and Salthouse (2017)	2,613	♂ y: 34% ♂ a: 28% ♂ o: 37% ♀ y: 66% ♀ a: 72% ♀ o: 63%	No clinic	United States	NR	18–99	Young adults (y) Adults (a) Older adults (o)	SNQ	WAIS; PNT; MCSAT; LPCT; DST; LST; SA; MR; FBT; PFT; SRT; LMT; FRT; PAT
Zuelsdorff et al. (2017)	1,052	♂: 31% ♀: 69%	No clinic	United States	White: 95% Non-white: 5%	40–78 M: 60	Middle-aged Older adults	MOS	RAVLT; BVMT-R; TMT-A; TMT-B; SC-WT; WAIS-III: DSF, DSB, LNS
Ge et al. (2017)	3,159	♂: 41% ♀: 59%	NR	United States (chinesepopulation)	NR	60–105 M: 73 SD: 8.3	Older adults	HRS	EBMT; BDST; SDMT
Zamora-Macorra et al. (2017)	2,211	♂: 46% ♀: 54%	NR	Mexico	NR	>50 M: 62 SD: 9.9	Adults Older adults	SNI; SCI; TI	MWS; VF; CERAD
Wang et al. (2017)	173	♂: 48% ♀: 52%	Peritoneal dialysis	China	NR	M: 55.5 SD: 12.2	Middle-aged Older adults	SSRS	3MS

NR, Not reported; M, Mean; SD, Standard Deviation; *, Homonyms but different instruments; For acronym's meanings, see section List of Nomenclatures.

#### Sample Characteristics of the Selected Studies

Sample size was ≤1,000 participants in 12 studies (54.5%) and was larger than 1,000 participants in 10 studies (45.5%). The study with the smallest sample had 120 participants (Zhu et al., 2012), while the largest sample had 13,119 participants (Kats et al., 2016). Most of the selected studies included participants older than 60 years in their sample, with the exception of three studies (13.6%) (Slykerman et al., 2005; Sims et al., 2011; Tanzer et al., 2013). Additionally, there were two studies (9.1%) that included participants between 18 and 105 years old (Yilmaz et al., 2015; La Fleur and Salthouse, 2017). Therefore, the reviewed articles focused on older adults (12 studies), middle adults (one study), young adults (one study) children (one study), and mixed-age groups (seven studies).

**Figure 3:**
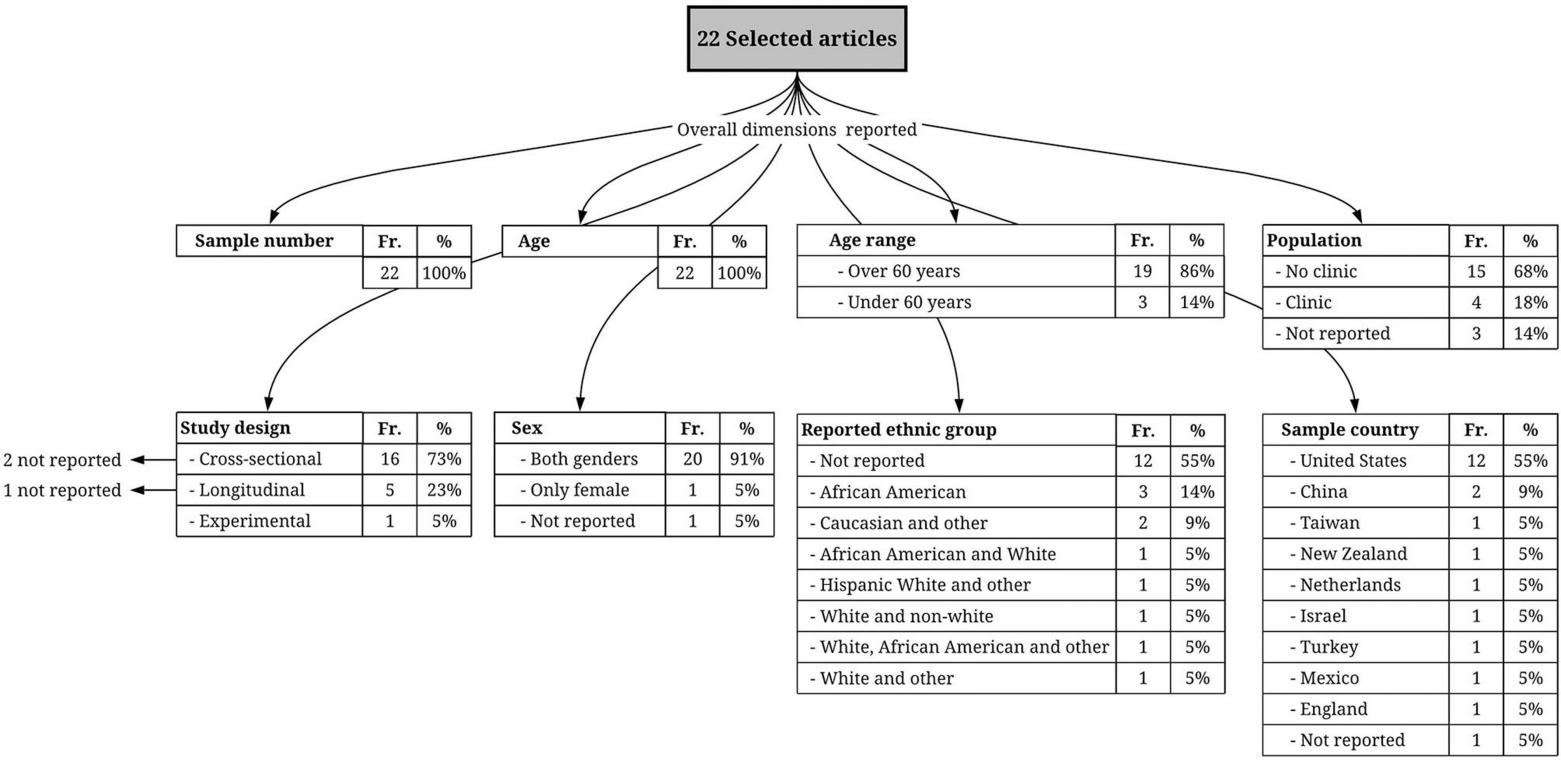
Flowchart representing methodological aspects of the selected studies; the frequency (Fr.) of each one of these aspects among the studies and the proportion (%) they represent within the total number of selected articles.

Most of the study samples were composed of both males and females, except one with only female participants (Tanzer et al., 2013). Only one study did not report information on gender (Slykerman et al., 2005). Many of the studies collected data from community samples, except four studies in which the population was composed of patients with depression (1 study, Dickinson et al., 2011), diabetes mellitus (1 study, Yilmaz et al., 2015), risk of atherosclerosis (1 study, Kats et al., 2016) or peritoneal dialysis (1 study, Wang et al., 2017). Only three studies did not report this information (Liao et al., 2016; Ge et al., 2017; Zamora-Macorra et al., 2017). Twelve studies were conducted with residents of the United States, two with residents of China, and one each with residents of New Zealand, Holland, Israel, Turkey, Mexico, and England, while one did not report residency information. In addition, 10 articles (45.5%) provided self-reported ethnicity, and 12 did not provide this information (54.5%). Finally, 16 studies used an observational cross-sectional research design, five used a longitudinal observational design (Seeman et al., 2001; Slykerman et al., 2005; Dickinson et al., 2011; Kats et al., 2016; Liao et al., 2016), and one used an experimental cross-sectional design (Tanzer et al., 2013). Of these, three studies did not explicitly report this information (Whitfield and Wiggins, 2003; Slykerman et al., 2005; Ayotte et al., 2013). For detailed information, see Table 3 and Figure 3.

### General Descriptive Analysis of the Results

Of the reviewed articles, 17 found a significant positive relationship between some or all of the social support factors they assessed (e.g., emotional support, perceived availability) and cognition; one study reported an effect of cognition on social support and not vice versa (Liao et al., 2016); two studies found a significant negative relationship between social support and cognition (Sims et al., 2014; Wang et al., 2017); and two studies found mixed results between these variables (Ayotte et al., 2013; La Fleur and Salthouse, 2017). For detailed information, see Table 4.

**Table 4:** Summary of analysis methods, variables and results.

**Author and Design**	**Analysis method (covariates)**	**Main results**
Seeman et al. (2001) Longitudinal	Bivariate and Multivariate (age, education, income, ethnicity, baseline health status, levels of physical activity, depressive symptoms, and self-efficacy beliefs)	- Greater frequency of emotional support exhibits significantly better cognition in transverse and longitudinal analyzes. It was the only social factor that independently related to the change in cognition in a follow-up 7.5 years later. This factor predicts better cognition, but its perceptual variation is small.
Yeh and Liu (2003) Cross-sectional	Multivariate (age, gender, religion, occupation, and health-condition variables)	- Higher cognition was clearly associated with married elders and those who perceived positive support from friends.
Whitfield and Wiggins (2003) Cross-sectional (NR)	Multivariate (age, gender, education, current health, chronic illnesses, physical limitations, SS) SEM (physical limitations)	- Greater SS perceived and given lead to higher levels of cognitive performance. - The mediation of the physical limitations was not completely responsible for the relationship between the SS and cognition.
Slykerman et al. (2005) Longitudinal (NR)	Multivariate (gestation, infant gender, maternal education, marital status, socioeconomic status, maternal age, parity, maternal smoking during pregnancy, duration of breastfeeding and examiner administering the Stanford Binet)	- SS during pregnancy was significantly associated with better cognition in children. - When analyzing the group of children with lower gestational weight at birth separately, no significant association was found with higher cognition.
Dickinson et al. (2011) Longitudinal	Bivariate and Multivariate (Sex, age, education, and participants' diagnostic status) (depressed or comparison participant)	- Lower social interaction was associated with lower cognition (CERAD and DSF) - Lower instrumental SS was associated with a decrease in cognition (ADS and SDMT)
Sims et al. (2011) Cross-sectional	Bivariate and Multivariate (age, gender, and education)	- Greater appraisal support, tangible support, self-esteem support, belonging support, and total support were significantly correlated with greater cognitive performance (SC-WT and WCST), and remained after controlling the covariates.
Zhu et al. (2012) Cross-sectional	Multivariate (age, gender, education, marital status, chronic diseases, income, residential arrangement)	- Family support, education, income, total SS and family support were significantly associated with cognition. - Demographic characteristics and SS together explained 45.2% of the variance in cognition.
Zuelsdorff et al. (2013) Cross-sectional	Multivariate (age, gender, education, and number of APOE ε4 alleles, smoking status, and marital/partner status)	- Higher SS index score was significantly associated with higher cognition (speed and flexibility) and remained in the full model.
Ellwardt et al. (2013) Cross-sectional	Multivariate (age, gender, level of education and physical functioning)	- Indirect association between emotional support and cognition, since emotional support was related to less loneliness, and less loneliness was associated with better cognition. However, there was also a significant direct relationship between emotional support and cognition. - Instrumental support is indirectly associated with cognition, but not directly. - Emotionally supportive relationships were stronger protectors against cognitive decline than instrumentally supportive relationships.
Tanzer et al. (2013) Experimental	Bivariate and Multivariate (depressive symptoms)	- Perceived SS being associated with cognition (recognition of happy facial expression, but not with angry expression). - A negatively directed association between perceived SS and recognition of an angry expression under the failure condition, but not under the success condition.
Ayotte et al. (2013) Cross-sectional (NR)	SEM [age, education, income, sex, and SF36 scores (overall health)]	- Age, functional limitations, and receipt of SS were negatively associated with cognition (fluid ability). - Education, income, and provision of SS were positively associated with cognition (fluid ability).
Sims et al. (2014) Cross-sectional	Multivariate (age, gender, education, depressive symptomatology, systolic blood pressure, body mass index, total cholesterol, and fasting glucose)	- No significant positive relations were found between SS and cognition in any domain. On the contrary, several functions of SS showed significant inverse relations with cognition, such that greater perceived SS was associated with poorer cognition (nonverbal memory and response inhibition).
Pillemer and Holtzer (2015) Cross-sectional	Bivariate and Multivariate (age, education, gender, and depression)	- The general level of perceived SS was positively associated with cognition. - The emotional/informational support factors were positively associated with higher cognition with and without control of covariates. - In a stratified correlation analysis between emotional / informational support and cognitive performance, a significant positive correlation was observed in women but not in men.
Yilmaz et al. (2015) Cross-sectional	Bivariate	- Overall SS score has significant and positive effects on cognition. - There was a significant relationship between SS (family support) and cognition (orientation and language subscales), and SS (significant others) and cognition (orientation, attention, memory and language subscales). - In accordance with the correlation analysis, the participants with cognitive dysfunction (CD) were determined to have significantly lower mean SS scores than those without CD. - The SS, especially from family and significant others, affected the development of CD in individuals with diabetes mellitus (DM).
Kats et al. (2016) Longitudinal	Multivariate (age, sex, study center, highest education level, cigarette smoking, alcohol consumption, prevalent hypertension and prevalent diabetes)	- SS (interpersonal support and social network) was associated with higher cognition in both racial groups, controlling for covariates, in mid-life. - There were no longitudinal effects between the variables of interest. - Higher level of SS was moderately associated with greater cognition at mid-life but did not predict change in global cognitive function in older adulthood.
Liao et al. (2016) Longitudinal	Bivariate and multivariate (Age, sex, ethnicity, longstanding illness, depressive symptoms, and prevalent chronic diseases, education, employment grades and marital history)	- Cognition modified SS (confiding and practical support) but not vice versa.
Frith and Loprinzi (2017) Cross-sectional	Multivariate (age, gender, race-ethnicity, measured body mass index, C-reactive protein, self-reported smoking status, self-reported diabetes status, measured mean arterial pressure, and self-reported physical activity)	- Those who received some type of support were associated with greater cognition than those who did not report SS. - The only individual source of support that was significantly associated with cognition was spouse-related support. - Those with a larger supportive network had greater cognition.
La Fleur and Salthouse (2017) Cross-sectional	Multivariate (age, sex, education and self-reported health)	- SS (social contact with family) significantly and negatively predicted cognition [vocabulary and g (global cognition or g score)]. - SS (social contact with friends, received emotional and anticipated perceived support) significantly and positively predicted all aspects of cognition and g. - Only social contact with family, received informational support, and provided emotional and informational support had any remaining significant relations with specific cognitive abilities. - Negative interactions significantly and negatively predicted all aspects of cognition and g.
Ge et al. (2017) Cross-sectional	Bivariate and Multivariate (age, gender, education, marital status, personal annual income, length of residence in the community, living arrangement, acculturation, depression, medical conditions and physical function)	- Older adults who received greater general SS tended to better preserve their overall cognition. - None of the sources of SS/strain was significantly associated with working memory.
Zuelsdorff et al. (2017) Cross-sectional	Multivariate [age, gender, race, education, APOE ε4 carrier status, parental history of Alzheimer's Disease (AD), Wisconsin Registry for Alzheimer's Prevention (WRAP) clinic site, smoking history and physical activity age and partner status]	- Higher SS scores were associated with more cognition (Speed & Flexibility and Immediate Memory) controlling for demographic variables. The significant relationship with immediate memory was lost when the partner status was incorporated. - Verbal interaction showed positive associations with both cognitive functions (Speed & Flexibility and Verbal Learning & Memory) controlling for demographic variables. The relationship between interaction quantity and Speed & Flexibility ceased to be significant when the quality of the interactions was controlled.
Zamora-Macorra et al. (2017) Cross-sectional	Bivariate and Multivariate (Sex, age, marital status, place of residency, education, and household members)	- Respondents with less support showed a greater chance of developing cognitive impairment than those with higher support.
Wang et al. (2017) Cross-sectional	Multivariate [age, gender, education, body mass index (BMI), diabetes, cardiovascular disease, serum albumin, High-sensitive C-reactive protein (hs-CRP) and total Kt/V]	- Higher global SS was associated with a higher risk of cognitive impairment, in an analysis with covariate adjustment. - SS had a significant negative association with cognitive function, especially subjective support.

*SS, Social Support; NR, Not reported; Articles sorted by year of publication. For acronym's meanings, see section List of Nomenclatures*.

### Social Support Measures

The majority of the studies used standardized self-report questionnaires (Slykerman et al., 2005; Sims et al., 2011, 2014; Zhu et al., 2012; Tanzer et al., 2013; Zuelsdorff et al., 2013, 2017; Pillemer and Holtzer, 2015; Yilmaz et al., 2015; Kats et al., 2016; Liao et al., 2016; Wang et al., 2017; Zamora-Macorra et al., 2017). However, seven studies used non-standardized surveys (Seeman et al., 2001; Yeh and Liu, 2003; Dickinson et al., 2011; Ellwardt et al., 2013; Frith and Loprinzi, 2017; Ge et al., 2017; La Fleur and Salthouse, 2017); and two studies used questions that were part of the National Survey on Black Americans (Whitfield and Wiggins, 2003; Ayotte et al., 2013).

Importantly, studies considered different dimensions of Social Support, as seen in Table 5. Most of the measures ask about sources of social support (also called structural support) and then distinguish between how often they can rely upon those sources (called availability in some studies), and how accessible they perceive these sources to be when talking about feelings (also called sharing of emotions, emotional support and intimacy) (Seeman et al., 2001; Yeh and Liu, 2003; Dickinson et al., 2011; Sims et al., 2011, 2014; Zhu et al., 2012; Ellwardt et al., 2013; Tanzer et al., 2013; Zuelsdorff et al., 2013, 2017; Pillemer and Holtzer, 2015; Yilmaz et al., 2015; Kats et al., 2016; Frith and Loprinzi, 2017; Ge et al., 2017; Wang et al., 2017).

**Table 5:** Summary of dimensions of social support's measures as named in the articles.

**Studies**	**Measures name**	**Dimensions**
Seeman et al. (2001)	MacArthur Battery (MAB)	(1) Quantitative/structural features, (2) types of ties, (3) instrumental social support, (4) emotional social support, (5) sources of demands and criticism
- Whitfield and Wiggins (2003) - Ayotte et al. (2013)	National Survey on Black Americans (NSBA)	Measures social support received and provided
Slykerman et al. (2005)	Family Support Scale (FSS)	Designed to assess how helpful different types of social support are to families rearing young children
Dickinson et al. (2011)	Duke Social Support Index (DSSI)	(1) Subjective social support, (2) instrumental social support, (3) social network size, (4) social interaction
- Sims et al. (2011, 2014)	Interpersonal Support Evaluation List (ISEL)	Self-report questionnaire that measures overall perceived social support and perceived availability in four dimensions: (1) belonging, (2) appraisal, (3) tangible, (4) self-esteem
- Zhu et al. (2012) - Yilmaz et al. (2015)	Multidimensional Scale of Perceived Social Support (MSPSS)	(1) Family support, (2) support from friends, (3) support from significant others
- Zuelsdorff et al. (2013, 2017)	Medical Outcomes Survey (MOS)	It measures social support perceived by people in times of need
Tanzer et al. (2013)	Network of Relationship Inventory (NRI)	Measures perceived social support from: (1) family, (2) close friend, (3) romantic partner, (4) other important figures
Pillemer and Holtzer (2015)	Medical Outcomes Study-Social Support Survey (MOS-SSS)	(1) Emotional support, (2) informational support, (3) tangible support, (4) affectionate support, (5) positive social interaction
Kats et al. (2016)	Short form of the Interpersonal Support Evaluation List (ISEL-SF)	(1) Appraisal support, (2) tangible assets, (3) belonging support, (4) self-esteem support
	Lubben Social Network Scale (LSNS)	Self-assessed measure of the active social network of: (1) family, (2) friends, (3) peers
Liao et al. (2016)	Close Person Questionnaire (CPQ)	(1) Confiding support (emotional support), (2) practical support, (3) negative aspects of close relationships
La Fleur and Salthouse (2017)	Social Network Questionnaire (SNQ)	(1) Social contact, (2) received support, (3) provided support, (4) perceived support
Ge et al. (2017)	Health and Retirement Study (HRS)	Social support was measured through the following sources: (1) spouse, (2) other family members, (3) friends
Zamora-Macorra et al. (2017)	- Social network index (SNI) - Social cohesion index (SCI) - Trust Index (TI)	The level of social support was calculated through three indicators: (1) Social network index (SNI), (2) Social cohesion index (SCI), (3) Trust Index (TI)
Wang et al. (2017)	Social Support Rating Scale (SSRS)	(1) Subjective support, (2) objective support, (3) support utilization
- Yeh and Liu (2003) - Ellwardt et al. (2013) - Frith and Loprinzi (2017)	NA	Series of questions

*NA, not applicable*.

The second-largest group of studies (Sims et al., 2011, 2014; Zuelsdorff et al., 2013, 2017; Pillemer and Holtzer, 2015; Kats et al., 2016) considers more dimensions of Social Support. They draw distinctions between emotional support, tangible support (also named objective, instrumental, practical), informational support, affectionate support, self-esteem, belonging, appraisal, and positive social interactions.

Only two studies (Liao et al., 2016; La Fleur and Salthouse, 2017) considered the dimension of negative aspects of social interactions. The support received from public services was measured only by three studies (Whitfield and Wiggins, 2003; Ayotte et al., 2013; Zamora-Macorra et al., 2017). Finally, only two studies assessed social support provided to others (Whitfield and Wiggins, 2003; Ayotte et al., 2013). The social support measures are detailed below (see also Tables 3, 5).

#### Standardized Self-Report Scales of Social Support

Three studies (Sims et al., 2011, 2014; Kats et al., 2016) used the Interpersonal Support Evaluation List (ISEL; Cohen et al., 1985), a questionnaire measuring perceived social support and the availability of four specific dimensions: belonging, appraisal, tangible support, and self-esteem. The Medical Outcome Study Social Support Survey (MOSS; Sherbourne and Stewart, 1991) was also used by three studies (Zuelsdorff et al., 2013, 2017; Pillemer and Holtzer, 2015). Similar to the ISEL, the MOSS assesses emotional support, informational support, tangible support, affectionate support and positive social interactions. In line with the mentioned tools, two studies (Zhu et al., 2012; Yilmaz et al., 2015) used the Multidimensional Scale of Perceived Social Support (MSPSS; Eker et al., 1995) and one study (Kats et al., 2016) used the Lubben Social Network Scale (Lubben and Gironda, 2004). Both questionnaires evaluate the availability of social support sources (friends, relatives and neighbors) by asking about quantity (i.e., “How many people”) and quality (i.e., “How often”).

The remaining studies (Slykerman et al., 2005; Tanzer et al., 2013; Liao et al., 2016; Wang et al., 2017; Zamora-Macorra et al., 2017) each use a different scale, respectively: The Close Person Questionnaire (Stansfeld and Marmot, 1992); The Family Support Scale (Dunst, 1984); The Network Relationship Inventory (Furman and Buhrmester, 1985); The Social Support Rating Scale (Xiao, 1994); The Social Network Index (Heaney and Israel, 2008) together with the Social Cohesion Index (Ramlagan et al., 2013) and the Trust Index (Zamora-Macorra et al., 2017). The dimensions of social support considered in these measures are detailed in Table 5.

#### Non-standardized Surveys

Five studies assessed social support by asking the participants questions without using a formal measure (Seeman et al., 2001; Yeh and Liu, 2003; Ellwardt et al., 2013; Frith and Loprinzi, 2017; Ge et al., 2017). They asked about sources of social support (e.g., friends, spouse, other family members) and perception of the availability of these sources (e.g., “How often can you rely on -a source of social support- for help if you have a problem?”).

#### National Survey on Black Americans

Two studies (Whitfield and Wiggins, 2003; Ayotte et al., 2013) used a data set from a large national study. In the survey, participants were asked how often they received different types of support (companionship, advice, financial assistance, among others). Interestingly, this survey asked about the frequency of providing social support to others.

### Cognition Measures

The majority of studies used a combination of different psychometric tests to measure a range of cognitive abilities. In general, three groups of measures can be identified across the reviewed literature: studies focusing on level of cognitive performance; those oriented at cognitive decline and one study using an experimental task.

#### Cognitive Performance

One group of studies focused on measuring cognition by assessing performance levels in several cognitive functions (i.e., memory, attention, reasoning). Among them, the majority (Dickinson et al., 2011; Ayotte et al., 2013; Zuelsdorff et al., 2013, 2017; Sims et al., 2014; Kats et al., 2016; Frith and Loprinzi, 2017; La Fleur and Salthouse, 2017; Zamora-Macorra et al., 2017) either totally or partially used the Wechsler Adult Intelligence Scale (WAIS; Wechsler, 1981), which measures intelligence and cognitive abilities.

Similarly, one study (Sims et al., 2011) used the Wisconsin Card Sorting Test (WCST; Grant and Berg, 1948) together with the Stroop Color and Word Test (SCWT; Stroop, 1935) which are considered measures of multiple cognitive functions (Scarpina and Tagini, 2017). Finally, the study by Slykerman et al. (2005) used the Stanford-Binet Intelligence Scale (Thorndike et al., 1986) to assess children's cognitive performance.

#### Cognitive Decline/Impairments

Several studies were focused on cognitive decline or cognitive impairments (Seeman et al., 2001; Dickinson et al., 2011; Zhu et al., 2012; Ellwardt et al., 2013; Yilmaz et al., 2015; Ge et al., 2017; Wang et al., 2017). The majority of them applied the Mini-Mental State Examination (MMSE; Folstein et al., 1975) a questionnaire widely used to measure cognitive impairment. Other studies (Whitfield and Wiggins, 2003; Yeh and Liu, 2003; Pillemer and Holtzer, 2015; Liao et al., 2016) used different measures to trace cognitive decline: the Alice Heim Group Ability Test (AH4; Heim, 1970); the Repeatable Battery for the Assessment of Neuropsychological Status (RBANS; Randolph et al., 1998); the Short Portable Mental Status Questionnaire (SPMSQ; Pfeiffer, 1975); the Everyday Problems Test (EPT; Willis et al., 1992), and the Boston Naming Test (BNT; Goodglass et al., 1983).

#### Experimental Cognitive Task

Tanzer et al. (2013) was the only study that used an experimental task to measure cognition. They tested the modulational role of perceived social support on recognition of facial expressions (i.e., angry vs. happy) in a computational task. Failure or success was induced experimentally before the task's execution with the aim of affecting cognitive performance.

### Relationship Between Social Support and Cognition

**Figure 4:**
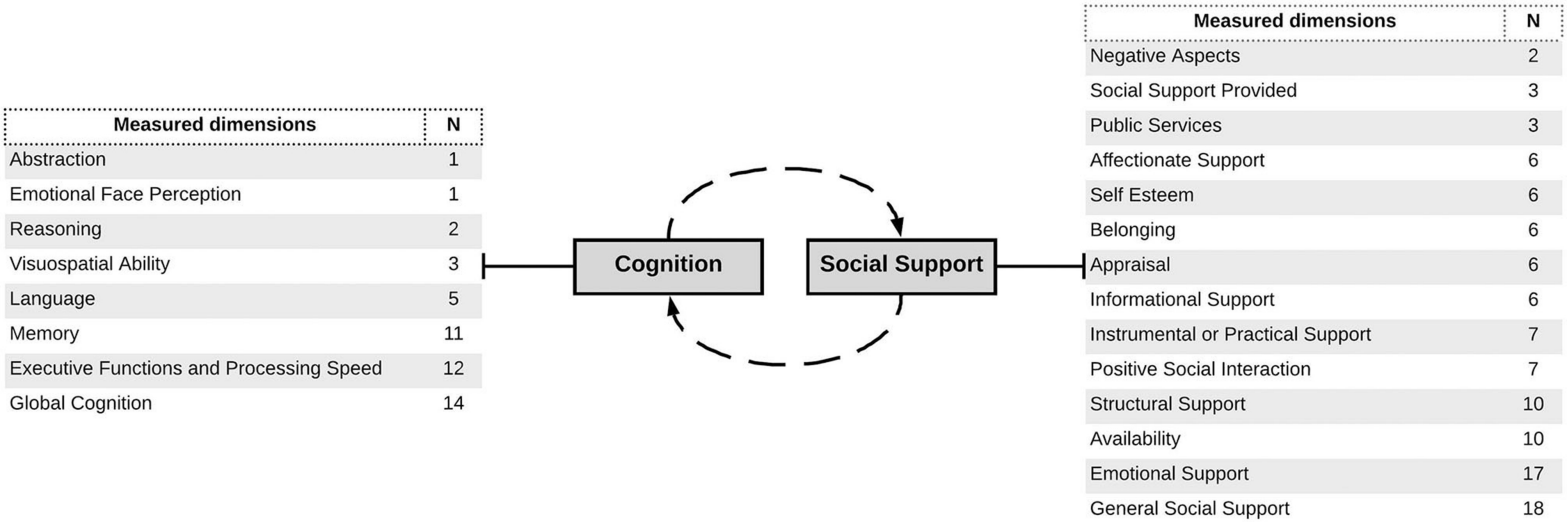
Flowchart representing the dimensions measured in both social support and cognition. The table on the left shows dimensions of cognition measured across the selected studies and the frequency with which each of them was tested across the studies (*N*). The table on the right shows the same process regarding dimensions of social support. Please note that categories were created by the authors of the present review by clustering similar dimensions across the studies.

Regarding the relationships between social support and cognition, the majority of articles reviewed found they were at least partially positively related. Of the 22 studies, only two found a negative relationship and two found mixed results between the variables. The results of the studies are summarized below, categorized according to their target age group due to the high proportion of studies that focused on a specific age group. A separate group was composed of studies on patients with chronic illnesses. This led to the identification of five categories (childhood, young and middle adulthood, older adulthood, more than one age group and chronic diseases). For the sake of clarity, Figure 4 summarizes the dimensions of both cognition and social support measured in the reviewed literature. Additionally, Table 6 presents the associations found in the studies between social support and cognition by dimensions.

**Table 6:** Summary of associations found between social support and cognition by dimension.

**Positive relationship**		**Results**
**Social support dimension**	**Cognition dimension**	
General social support	Global cognition	Pillemer and Holtzer, 2015; Kats et al., 2016; Frith and Loprinzi, 2017; Ge et al., 2017; Whitfield and Wiggins, 2003; Slykerman et al., 2005; Yilmaz et al., 2015; Zhu et al., 2012; Zamora-Macorra et al., 2017
	Executive functions and processing speed	Sims et al., 2011; Zuelsdorff et al., 2013, 2017
	Memory	Zuelsdorff et al., 2017
	Emotional face perception	Tanzer et al., 2013
Emotional support	Global cognition	Ellwardt et al., 2013; La Fleur and Salthouse, 2017; Seeman et al., 2001; Pillemer and Holtzer, 2015
Positive social interaction	Global cognition	Yeh and Liu, 2003; Kats et al., 2016
	Memory; executive functions and processing speed	Zuelsdorff et al., 2017
Instrumental or practical support	Memory; executive functions and processing speed	Dickinson et al., 2011; Sims et al., 2011
Availability	Global cognition	Dickinson et al., 2011; La Fleur and Salthouse, 2017
	Memory	Dickinson et al., 2011
Social support provided	Global cognition	Whitfield and Wiggins, 2003
	Executive functions and processing speed	Ayotte et al., 2013
Structural support	Global cognition	Kats et al., 2016; Frith and Loprinzi, 2017
Self esteem; belonging; appraisal	Executive functions and processing speed	Sims et al., 2011
**Negative relationship**		**Results**
**Social support dimension**	**Cognition dimension**	
General social support	Global cognition	Wang et al., 2017
	Executive functions and processing speed	Ayotte et al., 2013; Sims et al., 2014
	Memory	Sims et al., 2014
Availability	Global cognition	La Fleur and Salthouse, 2017; Wang et al., 2017
	Language	La Fleur and Salthouse, 2017
**Inverse relationship**		**Results**
**Social support dimension**	**Cognition dimension**	
Emotional support	Global cognition	Liao et al., 2016

*This analysis used the dimensions of Figure 4, and it was developed regardless of sociodemographic characteristics*.

#### Childhood

The research conducted by Slykerman et al. (2005) was the only one focused on infancy. They aimed to analyze the effect of social support received by mothers on their infant's cognition. They found that the social support received by mothers during pregnancy was significantly associated with cognition in infants whose birth weight was appropriate for their gestational age (Slykerman et al., 2005).

#### Young and Middle Adulthood

In the 19–57 age range, four studies found positive associations between some dimensions of social support and cognition. Kats et al. (2016) found a positive correlation between interpersonal support and cognition in their total sample but with gender differences: social network was associated with cognition in females but not in males. Similarly, in a sample composed of only female participants, Tanzer et al. (2013) found a positive association between social support and accuracy of facial expression recognition. Sims et al. (2011) found that dimensions of social support such as belonging, self-esteem, appraisal and tangible support predict cognition (executive functioning); they predict it both as independent dimensions and as a total factor. They argued these findings demonstrate the positive influence of social support on cognition prior to old age. In a study of middle-aged adults with a family history of Alzheimer's, Zuelsdorff et al. (2013) found greater social support was related to better performance in speed and flexibility but surprisingly, was not associated with memory performance.

#### Older Adulthood

The majority of the reviewed studies (Seeman et al., 2001; Whitfield and Wiggins, 2003; Dickinson et al., 2011; Zhu et al., 2012; Ayotte et al., 2013; Ellwardt et al., 2013; Sims et al., 2014; Pillemer and Holtzer, 2015; Kats et al., 2016; Liao et al., 2016; Frith and Loprinzi, 2017; Ge et al., 2017; Zamora-Macorra et al., 2017) involved an investigation of the association between social support and cognition in older adults. Among them, only Sims et al. (2014) found a negative relationship between social support and cognition, while Ayotte et al. (2013) found mixed results, and Liao et al. (2016) reported an effect of cognition on social support and not vice versa, whilst all the remaining studies found a positive association. We review these findings in detail below.

Emotional support was found to be a key dimension in the relationship between social support and cognition in three studies (Seeman et al., 2001; Ellwardt et al., 2013; Pillemer and Holtzer, 2015). Indeed, in a study conducted by Seeman et al. (2001), emotional support was the only social support dimension that predicted cognition in a follow-up study seven and a half years later. Pillemer and Holtzer (2015) found similar results but with important gender differences. In their study, perceived emotional support was significantly higher in females than in males and was also only positively associated with cognition in females. The authors argued that men and women use and experience social support differently, as women's networks are more multidimensional and robust than men's.

Similarly, the research conducted by Ellwardt et al. (2013) suggested that emotionally supportive relationships were stronger protectors against cognitive decline than instrumentally supportive relationships. On the contrary, Dickinson et al. (2011) found that instrumental support had an important role in predicting cognitive decline. They found that a decrease in social interaction and instrumental social support predicted a decline in cognitive performance and that this association was maintained after controlling for confounding variables. In the same way, Zamora-Macorra et al. (2017) also found that less social support is related to a greater probability of developing cognitive deterioration. Another social support factor found to be relevant was the role of the family. Zhu et al. (2012) found that social support (together with age and education) explained 45.2% of the variance in cognition, with family support being the strongest cognition predictor. Similarly, two studies reported that being married was associated with better cognition than being single (Yeh and Liu, 2003; Frith and Loprinzi, 2017). Contrarily, Seeman et al. (2001) found the unmarried state to be associated with higher cognition, especially in women. The authors explained this finding by considering that the married women in their sample tended to have older spouses who required care, a potentially stressful experience that might have a negative effect on cognition. Ayotte et al. (2013) found that increased reception of social support was negatively associated with cognitive performance, but that increased provided social support was positively associated with cognition. Similarly, Sims et al. (2014) did not find positive relationships between social support and cognition in any domain, but rather that higher perceived social support was associated with lower cognition (i.e., nonverbal memory and response inhibition). Additionally, in a longitudinal study conducted by Kats et al. (2016), social support was found to be a predictor of higher cognition in middle adulthood, but not in older adulthood. Finally, one study reported an effect of cognition on social support and not vice versa (Liao et al., 2016).

#### More Than One Age Group

Two studies focused on more than one age group. In La Fleur and Salthouse (2017), participant ages ranged from 18 to 99 years old. Results found an association between social contact with friends and emotional support with global cognition; understood as the sum or mean of different cognitive functions measured separately. Interestingly, in their study, contact with family showed a negative association with cognition. They explained this unexpected finding in terms of the potentially negative effect that interactions with family might imply. Similarly, Zuelsdorff et al. (2017) found a positive association between verbal interaction with other people and cognition in a sample composed of middle-aged and older adults. In their study, higher social support was related with better response velocity, flexibility, and immediate memory.

#### Chronic Illnesses

Two studies explored the role of social support on cognition in people with chronic illnesses with contradictory findings. Wang et al. (2017) used data from patients with peritoneal dialysis. The results showed higher global social support and that subjective social support predicted higher prevalence of cognitive impairment, whilst higher levels of independence were related with better immediate and delayed memory. They discussed these unexpected findings in terms of the potentially stressful experience that receiving social support might mean for some individuals, especially in the context of chronic illnesses. In contrast, in a study of patients with diabetes, Yilmaz et al. (2015) found a positive association between family support and cognition (i.e., language and orientation) and between the perception of support from significant others and subscales of orientation, attention, memory and language.

## Discussion

The purpose of this systematic review was to investigate the evidence from 1999 to 2019 regarding the relationship between social support and cognition. Among our findings, a relevant aspect was the significant increase in scientific interest in this topic in the last 11 years of the reviewed literature (Figure 2). The growing number of publications on the topic and the groundbreaking research (Ellwardt et al., 2013; Kats et al., 2016; Ge et al., 2017) confirm its relevance. Our results show a clear tendency to a positive relationship between social support and cognition across the studies reviewed, much higher than negative associations between the two or mixed results. This trend may imply that higher levels of social support positively affect people's cognitive functioning in different periods of their lives, even indirectly during the gestational period (Slykerman et al., 2005). However, the negative associations found may suggest the relationship between social support and cognition changes depending on the context (for example, in chronic illnesses; Wang et al., 2017).

Our first aim was to describe how social support was measured. In general, the tools used by the reviewed studies made the classical structural (i.e., an individual's social network characteristics) vs. functional (i.e., the perceived availability of different types of help) support distinction (Uchino et al., 2018). They also considered the emotional dimension of support to be separate from tangible forms of support, as well as the difference between perceived and received social support. The latter is a crucial distinction because perceived social support (i.e., a sense of having people to count on for help if needed) has shown to have a more significant influence on health than objective support (i.e., the received help) (Uchino et al., 2012). Only two studies (Liao et al., 2016; La Fleur and Salthouse, 2017) included negative aspects of social interactions in their social support measure, which is surprising because the potentially upsetting aspects of supportive relationships (i.e., feeling useless, controlled or in debt) have been consistently considered in the relation between social support and health in the literature. Such aspects are considered to be an essential dimension of social support because of their known negative influence on health outcomes (Barrera, 1986; Uchino et al., 2018), such as higher blood pressure and greater inflammation response (Uchino et al., 2012). Another dimension that was not adequately considered by the social support measures was the role of providing support to others, which was only included in two studies (Whitfield and Wiggins, 2003; Ayotte et al., 2013). This dimension reflects the fact that social support is not an artifact owned by either the receiver or provider. Instead, it appears that social support emerges within social interaction (Gottlieb and Bergen, 2010). Therefore, the dimensions of negative aspects of social interactions and the role of providing social support on cognition remain mostly unexamined. Finally, the wide variety of approaches used to measure social support across the literature, ranging from established validated scales to more arbitrary un-validated measures is striking. Although there is no gold-standard social support measure (Goodger et al., 1999; Chronister et al., 2006), future research should consider adopting validated measures in order to increase consensus and validity.

In regard to our second aim, measures of cognition, all the studies measured cognitive functioning with performance-based standardized instruments. Among them, we found three groups: (i) studies focusing on level of cognitive performance; (ii) one study using an experimental task; (iii) studies focused on cognitive decline.

Studies measuring cognitive performance tested a variety of skills and abilities (e.g., attention, language, memory) using a combination of psychometric tests. This openness may reflect an exploratory phase in the research about cognition and social support as it implies seeking associations without a hypothesized model of relationships with specific cognitive abilities. Another surprising finding in this regard was the lack of consideration of social cognitive factors among the array of cognitive capacities measured. From a theoretical point of view on the relationship between social support and cognition (Lakey and Drew, 1997; Lakey and Orehek, 2011), social cognition is a key cognitive process. However, among the studies reviewed, only one study (Tanzer et al., 2013) considered social cognition during a computational task on facial expression recognition. Regarding the group of studies measuring cognitive decline, they primarily used neuropsychological batteries, which may express a focus on clinical research. In that sense, the instruments used and the variety of skills assessed is coherent with the setting in which they were developed, where the goal is to investigate potential risk factors for neuropsychological deterioration or cognitive impairments.

Our results show that cognitive processes explored in relation to social support are very broad. In general, the studies reviewed fail to operationalize complex cognitive processes properly, which is a common problem in the study of cognition (Poeppel, 2012; Poeppel and Adolfi, 2020). Specifically, in the reviewed literature cognitive processes were usually defined in isolation from socio-cognitive factors, social context, social interaction, and/or ecological niche. The way they assess cognition implies a lack of consideration of the fact that neurocognitive structures are functionally coupled to and actively participate in larger brain/body/world cognitive arcs (Clark, 2017; Krakauer et al., 2017; Parada and Rossi, 2018). The measures used to assess cognitive abilities can be interpreted as assuming thought as a product of a mind that has rich internal representations of the external world. In this regard, we think it is important to consider recent theoretical and empirical work (Clark, 2017; Krakauer et al., 2017; Gallagher, 2018; Azzalini et al., 2019; Palacios-Garcia and Parada, 2019; Shamay-Tsoory and Mendelsohn, 2019; Parada and Rossi, 2020) conceptualizing cognition as a myriad of processes that, in order to promote successful adaptation to the world, can dynamically reconfigure their own boundaries. For example, mirror neurons were once thought of as genetically-fixed functional units (Gallese et al., 2009). Evidence acquired in the last decade suggests otherwise (see Cook et al., 2014). We now know these networks develop and change their functional properties through active engagement in sensorimotor learning. Thus, mirror neurons are a functional component of greater cycles of embedded perception-action arcs including dynamic body morphology mapping, action observation/production, object manipulation, offloading mental processes, among others.

Furthermore, such ideas have challenged the understanding of cognitive states and with that, the methods employed to assess them (Ladouce et al., 2017; Shamay-Tsoory and Mendelsohn, 2019; Parada and Rossi, 2020). The implementation of these novel ideas as empirical work is still fledgling, as novel methods are developed (Ladouce et al., 2017; Shamay-Tsoory and Mendelsohn, 2019), innovative experimental designs suggested (Parada, 2018; Matusz et al., 2019; Shamay-Tsoory and Mendelsohn, 2019), and neurocognitive scientists begin to discuss the implications of both epistemic and methodological advancements in a more profound manner (De Jaegher et al., 2016; Krakauer et al., 2017; Parada and Rossi, 2018, 2020; Buzsáki, 2020; Poeppel and Adolfi, 2020). The issues discussed above show that social support and cognition research is still in its infancy and could greatly benefit from these novel ideas.

The third aim of this review was to describe the reported relationships between social support and cognition. Most studies found a positive significant relationship between these variables. Some of them found total relationships (i.e., total scores of both measures) and others found partial relationships (i.e., between some factors of each measure, for example, between tangible support and memory). In this regard, the social support dimension that showed the strongest association with cognitive factors was emotional support. Emotional support (i.e., receiving nurturance from SS sources allowing the receiver to feel valued; Langford et al., 1997) was most frequently associated with cognition and was also supported by more robust evidence (i.e., longitudinal studies). This is coherent with current notions of emotion and cognition which conceive them to be part of the same phenomena (Hoemann and Feldman Barrett, 2018).

Regarding cognition, the so-called Global Cognition (GC; see Table 6) was the dimension most frequently associated with social support. GC is a score composed of a combination of separately measured cognitive functions. Although the composition of this total score varied among studies (restricting comparison), this finding can be understood by considering the influence of social interactions on cognition as a global phenomenon (Clark, 2017; Gallagher and Allen, 2018; Kirchhoff et al., 2018). Another interesting finding is that the majority of the reviewed studies (86%; Figure 3) involved an investigation of the effect of social support on cognition in older adults. This may reflect the increasing focus of research on the health consequences of aging due to the growth of this population, which is a public health concern worldwide (Beard et al., 2016). In this sense, the focus on older age groups may also reflect that the line of research on social support and cognition is mostly focused on clinical aspects of this association and less focused on basic psychological processes.

There were also unexpected findings across the reviewed literature. Sims et al. (2014) and Wang et al. (2017) found a negative relationship between social support and cognition, whilst Ayotte et al. (2013) and La Fleur and Salthouse (2017) found negative associations between some of the dimensions measured. However, these studies present important limitations regarding sample composition, making it difficult to extrapolate their results. For example, the study by Wang et al. (2017) focused specifically on patients under peritoneal dialysis and the Sims et al. (2014) sample is from a specific hospital and composed of highly educated people.

Finally, although some studies claimed to have found an effect of social support on cognition, the observational and cross-sectional nature of the majority of them restricts the inference of causality. This is an obvious limitation of the reviewed studies. However, despite limitations, there is overall preliminary evidence of a relevant positive association between social support and cognition. The results of the present review demonstrate there is enough information for an outbreak of experimental research in the area and an expansion of this body of knowledge. Having analyzed the results of this review, the following section addresses some of the main challenges and opportunities that this line of research currently faces.

### Challenges and Opportunities

The present review shows a clear increase in interest in social support (Figure 2) and some groundbreaking findings that reveal its importance for understanding human cognition in the context of social interaction. One of the main challenges entailed by the current literature on social support lies in the methodologies employed. First, the 22 studies reviewed used self-report methods, which has obvious reliability limitations. Additionally, some studies use un-validated social support measures. Second, there was a tendency toward too open hypotheses, with no previous models to understand the association between social support and cognition. The majority of the studies included a large number of both dependent and independent variables. Many researchers utilized both social support and cognition measures with numerous subscales and also examined multiple outcome variables, but without specifying any a priori hypotheses about the variables. If participant scores on each of the subscales were then tested for an association with all of the dependent variables, the total number of tests was often very large, and researchers rarely corrected for the number of tests conducted. Testing such a large number of associations increases the possibility of obtaining at least one significant finding by chance, amplifying Type I error. At best, this may lead to vague conclusions.

Even though the absence of experimental data restricts us from drawing robust conclusions, there is already enough evidence to test more specific models using experimental designs, which presents an opportunity. The evidence reviewed here provides an opportunity to lay the foundations for a more comprehensive theoretical model, one that corresponds with the complexity of the topic and possibly consider models derived from social interaction (Di Paolo and De Jaegher, 2012) and/or active inference (Gallagher and Allen, 2018; Kirchhoff et al., 2018).

### Strengths and Limitations of the Present Study

The inclusion of a number of observational studies precluded traditional meta-analysis. Instead, we adopted a narrative synthesis approach, which has several limitations. Appraisal of quality is difficult with such a variety of study designs, and data extraction relies heavily on the reviewers' interpretation of the literature, which may introduce bias. However, a narrative approach allows the synthesis of diverse literature into common themes relevant to the research question.

Another source of weakness in this study which could have affected its comprehensiveness was the use of the search terms only in title and summary. Although this strategy allowed us to focus on the more relevant studies (i.e., those expressly referred to the concepts of interest), a broader search might have resulted in a more exhaustive review.

Finally, a disadvantage of many systematic reviews is that even using multiple databases, it is highly likely that some relevant articles will be missed in the search (Bramer et al., 2017). In the present study, we found a good example of this limitation during the review process: One of the reviewers noted a missed article on the subject (Kotwal et al., 2016) that was not recalled in our search (as can be noticed in our OSF database https://osf.io/wbzfk/ where the whole process of search is detailed).

Nonetheless, this systematic review has performed its role in building a body of evidence from which to establish further enquiry.

## Conclusion

The present review has shown that the link between social support and cognition is a topic of increasing scientific interest with relevant groundbreaking findings, which demonstrate the need to start analyzing these issues in more detail. The absence of experimental data restricts the extraction of robust conclusions but the present evidence on the topic demonstrates that the field is prepared to move toward the next level.

## Data Availability Statement

The original contributions presented in the study are included in the article/supplementary material, further inquiries can be directed to the corresponding author/s.

## Author Contributions

SC-C and CA-R perform the searching process and organized the databases. SC-C, CA-R, and AR wrote the first draft of the manuscript. All authors contributed to conception and design of the study and revised the manuscript, read, and approved the submitted version.

## Conflict of Interest

The authors declare that the research was conducted in the absence of any commercial or financial relationships that could be construed as a potential conflict of interest.

## List of Nomenclatures

**Table:** Acronyms for each instrument mentioned in Tables 3, 4.

**Nomenclature (A-M)**	**Meaning**
ADS	Ascending Digit Span
AH4-I	Alice Heim 4-I test
AST	Alpha Span Test
BD	Block Design
BDST	Backwards Digit Span Test
BVMT-R	Brief Visuospatial Memory Test - Revised
CERAD	Consortium to Establish a Registry in Alzheimer's Disease
CP	Constructional Praxis
CPQ	Close Person Questionnaire
CT	Computerized Task
DB	Digits Backward
DF	Digits Forward
DOT	Digit Ordering Test
DSB	Digit Span Backward
DSF	Digit Span Forward
DSSI	Duke Social Support Index
DSST	Digit Symbol Substitution Test
DST	Digit Symbol Test
DWRT	Delayed Word Recall Test
EBMT	East Boston Memory Test
EPT	Everyday Problems Test
FBT	Form Boards Task
FRT	Free Recall Task
FSS	Family Support Scale
HRS	Health and Retirement Study
HVLT	Hopkins Verbal Learning Test
II-IV-ETS-VT	Parts II and IV of the ETS Vocabulary Test
IPT	Identical Pictures Test
IRT	Immediate Recall Task
ISEL	Interpersonal Support Evaluation List
ISEL-SF	Short form of the Interpersonal Support Evaluation List
JLO	Judgment of Line Orientation
LM-I	Logical Memory I
LM-II	Logical Memory II
LM-WMS-R	Logical Memory subtest of the Wechsler Memory Scale— Revised
LMT	Logical Memory Task
LNS	Letter-Number Sequence
LPCT	Letter and Pattern Comparison Task
LSNS	Lubben Social Network Scale
LST^*^	Letter Series Test
LST	Letter Sets Task
LT	Language Tasks
MAB	MacArthur Battery
MCSAT	Multiple-Choice Synonym and Antonym Task
MMSE	Mini Mental State Examination
MOS	Medical Outcomes Survey
MOS-SSS	Medical Outcomes Study-Social Support Survey
MR	Matrix Reasoning
MSPSS	Multidimensional Scale of Perceived Social Support
MWS	Memory Wechsler Scale
**Nomenclature (N-Z)**	**Meaning**
NCT	Number Comparison Test
NRI	Network of Relationship Inventory
NSBA	National Survey on Black Americans
OST	Operation Span Task
PAT	Paired Associates Task
PFT	Paper Folding Task
PNT	Picture-Naming Task
PVF	Phonemic Verbal Fluency
RAVLT	Rey Auditory Verbal Learning Test
RBANS	Repeatable Battery for the Assessment of Neuropsychological Status
RCPM	Raven Colored Progressive Matrices
SA	Shipley's Abstraction
SBIS-4E	Stanford Binet Intelligence Scale Fourth Edition
SC-WT	Stroop Color-Word Test
SCI	Social Cohesion Index
SDMT	Symbol Digit Modalities Test
SILSAT	Shipley Institute of Living Scale Abstraction Test
SILVMT	Shipley Institute of Living Verbal Meaning Test
SMMSE	Standardized Mini Mental State Examination
SNI	Social Network Index
SNQ	Social Network Questionnaire
SPMSQ	Short Portable Mental Status Questionnaire
SRT	Spatial Relations Task
SSRS	Social Support Rating Scale
SVF	Semantic Verbal Fluency
TA	Trails A
TB	Trails B
TI	Trust Index
TMT-A	Trail Making Test Parts A
TMT-B	Trail Making Test Parts B
TT	Trailmaking Test
VF	Verbal Fluency
VLM	Verbal Learning and Memory
VR-I	Visual Reproductions I
VR-II	Visual Reproductions II
VSB	Visual Span Backward
VSF	Visual Span Forward
WAIS	Wechsler Adult Intelligence Scale
WAIS-III	Weschler Adult Intelligence Scale–III
WAIS-R	Wechsler Adult Intelligence Scale—Revised
WCST	Wisconsin Card Sorting Test
WFT	Word Fluency Test
WMS-R	Wechsler Memory Scale—Revised
18I-BNT	18-item version of the Boston Naming Test
20-WAL	20-Word Audiotaped List
3MS	Mini-Mental State Examination modificado
4I-WAIS-R	4-items from the Similarities subtest of the Wechsler Adult Intelligence Scale—Revised

* *Homonyms but different instruments*.
